# A First‐In‐Human Randomized Controlled Phase 1 Study Assessing the Safety and Tolerability of Topical TCP‐25 Gel in Epidermal Suction Blister Wounds

**DOI:** 10.1111/cts.70497

**Published:** 2026-02-05

**Authors:** Karl Wallblom, Sigrid Lundgren, Ganna Petruk, Manoj Puthia, Jane Fisher, Matilda Hugerth, Karim Saleh, Artur Schmidtchen

**Affiliations:** ^1^ Division of Dermatology and Venereology, Department of Clinical Sciences Lund Lund University Lund Sweden; ^2^ Department of Dermatology Skane University Hospital Lund Sweden; ^3^ Division of Infection Medicine, Department of Clinical Sciences Lund Lund University Lund Sweden; ^4^ Xinnate AB Lund Sweden

**Keywords:** epidermolysis bullosa, inflammation, therapeutic peptide, wound exudation

## Abstract

Inflammation and infection remain unmet challenges in wounds of various etiologies, delaying healing, impacting quality of life, and increasing healthcare costs. The thrombin‐derived C‐terminal peptide TCP‐25 has demonstrated dual anti‐inflammatory and antibacterial activities in animal wound infection models, thereby promoting healing, highlighting its therapeutic potential as a wound treatment. This first‐in‐human, double‐blind, randomized, within‐person and placebo‐controlled clinical trial (NCT05378997) evaluated the safety, tolerability, and pharmacokinetics of topical TCP‐25 in healthy volunteers. Twenty‐four participants each received four suction blister wounds (two per thigh), with two wounds treated with TCP‐25 (either 0.86, 2.9, or 8.6 mg/mL) and two with placebo gel, 5 times over 8 days. For the primary safety endpoint, no serious or significant adverse events or withdrawals due to adverse events were reported. Twenty‐one participants (88%) reported at least 1 adverse event; all were mild or moderate and judged to be unlikely related to TCP‐25 treatment. No abnormal local reactions occurred and no clinically relevant changes in electrocardiogram, vital signs, laboratory parameters, or physical examination findings were observed between baseline and end of treatment. For the secondary endpoint, TCP‐25 was undetectable (< 90 nmol/L) in all plasma samples at all timepoints. Thus, topical TCP‐25 gel was safe and well tolerated by healthy volunteers with epidermal wounds, with no evidence of measurable systemic exposure. Exploratory analyses indicated reduced wound exudation with TCP‐25 treatment, particularly at 2.9 and 8.6 mg/mL. Taken together, these findings support further clinical evaluation of TCP‐25 in relevant patient populations.

## Study Highlights

- What is the current knowledge on the topic?
  ○ In preclinical models of wound infection, the immunomodulatory peptide TCP‐25 has consistently demonstrated dual anti‐inflammatory and antimicrobial activities that facilitate wound healing, underscoring its therapeutic potential.
  ○ TCP‐25 is being developed as a topical treatment for epidermolysis bullosa (EB) wounds.
  ○ However, at the time of this study, no safety, tolerability, or pharmacokinetic data in humans had been reported.
- What question did this study address?
  ○ This first‐in‐human study aimed to examine the safety, tolerability, and pharmacokinetics of TCP‐25 gel in healthy volunteers with suction blister‐induced wounds.
- What does this study add to our knowledge?
  ○ Topical application of TCP‐25 gel was safe and well tolerated in human volunteers with epidermal wounds, consistent with previous preclinical animal studies.
  ○ No systemic exposure to TCP‐25 was detected, and no new safety concerns were observed.
  ○ Exploratory analyses indicated that TCP‐25 reduced wound exudation.
- How might this change clinical pharmacology or translational science?
  ○ Based on its safety and tolerability profile, these findings support the further clinical development of TCP‐25 in wound treatment.
  ○ Pending evaluation in patients with disease‐related wounds that are more complex and difficult to treat, TCP‐25 may represent a novel treatment modality based on its multifaceted action on inflammation and bacteria.

## 1 Introduction

Wound healing is a complex, coordinated process that can be disrupted by factors such as infection, compromised vascular supply, or genetic predisposition, resulting in hard‐to‐heal wounds [1, 2]. Hard‐to‐heal wounds impose a substantial clinical burden, particularly in vulnerable populations such as the elderly, and are associated with serious complications including systemic infections, scarring, and malignant transformation [1, 3, 4].

Similar pathological challenges also affect younger patients, such as in the genetic skin disorder epidermolysis bullosa (EB). EB exemplifies how chronic wounding, persistent inflammation, and impaired wound healing can result in significant morbidity and early death due to wound‐related complications [3, 5, 6]. EB comprises a group of genetic disorders that manifest with blistering and fragility of the skin and other stratified epithelia, leading to a high wound burden [5, 7]. Hard‐to‐heal wounds in EB are characterized by excessive bacterial influence, particularly by *Staphylococcus aureus* , and dysregulated inflammation [8, 9, 10, 11]. Previous studies have linked these pathological features to poor clinical outcomes, including impaired wound healing [12] excessive exudation [13, 14] and skin cancer formation [3] as well as systemic complications such as chronic anemia and increased risk of sepsis [12, 15]. This underscores the urgent need for innovative therapeutics that can address the complex pathophysiology of wounds characterized by excessive inflammation and bacterial influence.

Thrombin‐derived C‐terminal peptides (TCPs) constitute a group of endogenous anti‐inflammatory and antimicrobial peptides of approximately 2–3 kDa that are naturally present in human wounds [16, 17, 18]. TCP‐25 is a synthetic 25‐amino acid peptide (GKYGFYTHVFRLKKWIQKVIDQFGE; 3088.6 Da) that corresponds to the C‐terminal region of human thrombin and encompasses several endogenous TCP sequences. Consequently, TCP‐25 can be cleaved into biologically active fragments similar to those naturally generated in wounds [18]. The dual anti‐inflammatory and antibacterial mode of action of TCP‐25 is well characterized, implicating its therapeutic potential as a wound treatment boosting the body's innate peptide‐based defense. With regard to its mechanisms, TCP‐25 downmodulates inflammation by binding to and neutralizing proinflammatory microbial products, such as lipopolysaccharide (LPS), lipoteichoic acid (LTA), peptidoglycan (PGN), lipid A, and zymosan [19, 20, 21, 22]. In addition, TCP‐25 binds with high affinity to the LPS‐binding pocket of CD14, the co‐receptor for TLR4, the main recognition receptor for LPS [19]. As a result of this interference, TLR4‐mediated inflammatory responses are reduced [19, 20, 23]. The antimicrobial mode of action of TCP‐25 is mediated by direct lysis of bacteria through membrane disintegration and permeabilization [16]. Preclinical studies have shown that TCP‐25 has antibacterial activity against the common wound pathogens *S. aureus* and *P. aeruginosa* and acts against several other gram‐positive and gram‐negative pathogens, including multiresistant bacterial strains [16, 18]. Proof‐of‐principle studies in porcine wound infection models have shown that a TCP‐25‐containing gel reduces inflammatory responses and treats *S. aureus* infection, improving wound healing [18].

Given its mode of action and demonstrated efficacy in reducing inflammation and infection while accelerating healing in experimental wound models, TCP‐25 is suited for broad therapeutic use in wounds where bacterial colonization and excessive inflammation impair healing, such as in EB wounds. However, due to the fragile skin of patients with EB, initial testing in this population is not feasible. Suction blister wounds were therefore selected for the first part of the Phase 1 study because they replicate epidermal detachment with exposed dermis, which is characteristic of EB blisters but without the underlying genetic defects.

In this first‐in‐human study, standardized epidermal wounds were induced in healthy volunteers using the suction blister technique. This method produces standardized wounds with consistent depth (through complete removal of the epidermal layer), size, and a predictable closure time. The resulting injury initiates a localized innate immune response, characterized by increased blood flow and infiltration of inflammatory cells into the exposed dermis. Over subsequent days, wounds typically become colonized by commensal bacteria [24, 25].

The primary aim of this study was to assess the safety and tolerability of escalating topical doses of TCP‐25 gel applied to suction blister wounds. The secondary aim was to assess systemic exposure and pharmacokinetics. Clinical parameters related to wound exudation were recorded and analyzed as exploratory outcomes.

## 2 Materials and Methods

The CONSORT reporting guidelines with extensions for dose finding, pharmacokinetics, safety, and within‐person randomization were used to prepare relevant parts of this manuscript [26, 27, 28, 29]. All research procedures were performed in strict accordance with a predefined protocol and adhered to the international good clinical practice guidelines, the Declaration of Helsinki, the European Union Clinical Trials Directive 2001/20/EC, and applicable local regulatory requirements. The Swedish Ethical Review Authority approved the study on 10 March 2022 (Etikprövningsmyndigheten, application number 2022‐00527‐01). All participants signed informed consent forms prior to participation.

This study is the first part of a 3‐part clinical trial registered at ClinicalTrials.gov under the identifier NCT05378997. The trial was prospectively entered into the EudraCT database on 16 November 2021 (EudraCT number: 2021‐004728‐14). The registration at ClinicalTrials.gov (NCT05378997) was submitted on 25 April 2022 and posted on 18 May 2022. Registration at ClinicalTrials.gov was retrospectively completed a few days late due to logistical delays, as reported in the published study protocol [30].

### 2.1 Trial Design

The study was designed as a double‐blind, randomized, within‐person, and placebo‐controlled first‐in‐human study. A within‐person study design reduces inter‐individual variability and sample size required, with wound sites standardized and randomized to reduce location bias. To avoid carryover effects, wounds were separated and dressed individually.

### 2.2 Participants and Setting

The study was performed at the Lund Clinical Trial Unit of Skåne University Hospital. Healthy volunteers were recruited from the clinic's database of healthy volunteers and by advertisement. The initial screening took place within 28 days prior to Day 1 and included an eligibility check and review of each participant's health status. Healthy volunteers aged 18 to 60 years who provided written informed consent and could comply with requirements were eligible. Detailed inclusion and exclusion criteria are provided in the Supporting Information. Participants could withdraw voluntarily at any time or be discontinued by the investigator for significant adverse events (AEs), severe protocol non‐compliance, loss to follow‐up, or prohibited medication use, as further described in the Supporting Information.

The plan was to screen up to 50 participants and ultimately randomize 24 participants to 3 study arms. The planned cohort size was determined based on the expected variability and in accordance with first‐in‐human study design principles. No formal power calculations were performed as the study was dimensioned for primary safety endpoints, with other parameters designated as exploratory outcomes.

Demographic information, baseline characteristics, medical/surgical histories, and concomitant medications were documented in electronic case report forms (eCRF) and coded using MedDRA (v24.1) and the WHO Drug Dictionary (2021). Definitions and data gathered are detailed in the Supporting Information. Data management and monitoring followed established quality control procedures (described in Supporting Information).

### 2.3 Randomization and Blinding

**FIGURE 1:**
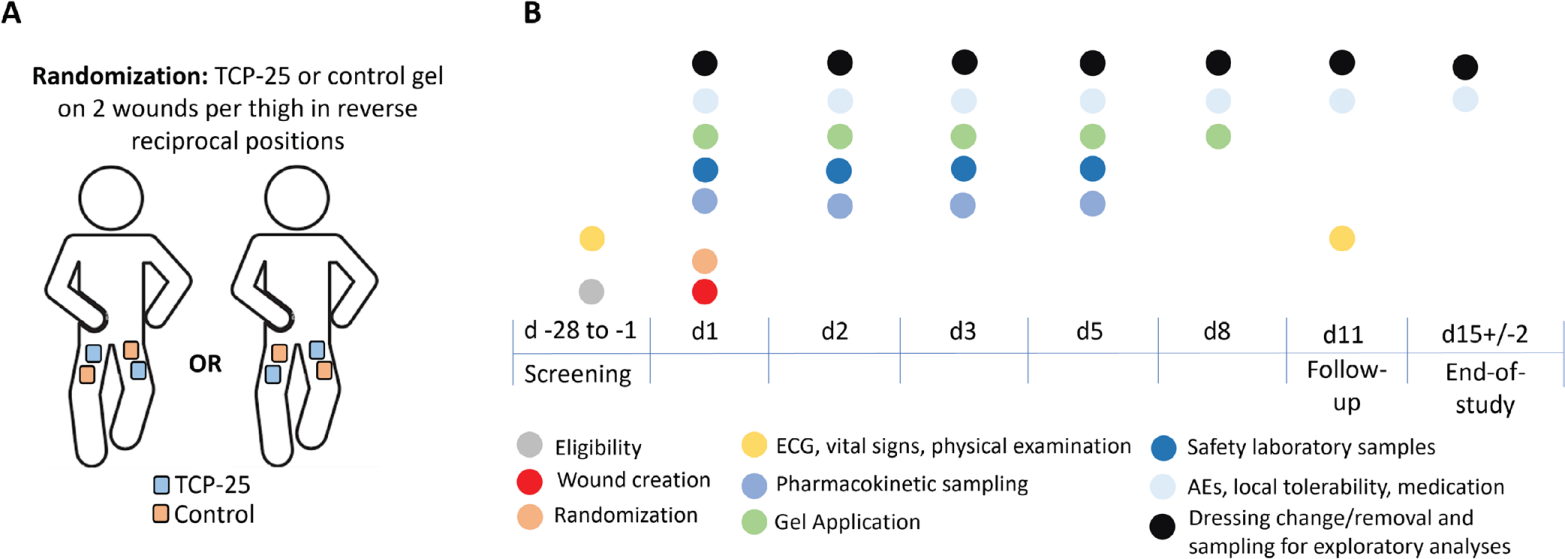
Randomization and study schedule. (A) Schematic representation of the wounds and the randomized treatment allocation. (B) Schedule of treatments and assessments. The participants attended the clinic for 8 visits in total for screening, wound creation, treatment, and follow‐up. Treatment was administered a total of 5 times, with adverse reactions, including local tolerability assessments, evaluated 7 times.

Participants were randomized using a computer‐generated allocation list prepared with SAS software (v9.4, SAS Proc Plan) by a designated staff member at Clinical Trial Consultants AB. Randomization assigned two wounds per participant (one proximal and one distal, on opposite thighs) to TCP‐25 and placebo, ensuring balanced allocation (Figure 1A). Participants, caregivers, investigators, clinical staff, and outcome assessors remained blinded to treatment assignments, facilitated by the identical appearance of TCP‐25 and placebo gels. Treatment allocations remained concealed until after database lock. The practical details for handling randomization are outlined in the Supporting Information.

### 2.4 Intervention and Treatment Administration

On Day 1, the participants visited the clinic for wound creation. Epidermal wounds (0.8 cm^2^) were formed using the suction blister technique as described by Larsen et al. [31] and in the published protocol [30]. This procedure resulted in four suction blister wounds per participant, with two wounds on each thigh (Figure 1A). Prior to blister induction, the area was gently shaved according to established procedures [31, 32].

TCP‐25 and placebo gels were formulated as proprietary hydrogels based on hydroxyethyl cellulose, with glycerol for isotonicity (pH 7.0). The placebo gel was identical but lacked TCP‐25. TCP‐25 was administered at concentrations of 0.86, 2.9, and 8.6 mg/mL. The TCP‐25 gel was manufactured in accordance with good manufacturing practice as a sterile investigational medicinal product by a licensed contract manufacturer and was supplied by Xinnate AB. The schedule for treatment administration and assessment is shown in Figure 1B.

TCP‐25 was evaluated in three sequential dose groups (*n* = 8 participants/group, total *n* = 24), each receiving topical administration on four thigh wounds per participant (two TCP‐25, two placebo, randomized allocation). Each wound received five topical applications over 8 days (Days 1, 2, 3, 5, and 8).

Gel application (0.15 mL) was performed using 1‐mL syringes, placing the gel centrally onto each wound and immediately covering it with a 2 × 2‐cm polyurethane foam dressing (Mepilex Transfer, Mölnlycke Healthcare, Gothenburg, Sweden). Gentle pressure was applied to evenly distribute the gel across the wound surface, including the skin edges, followed by the application of a secondary transparent dressing (Tegaderm, 3M, Saint Paul, USA) and a protective gauze pad secured with additional Tegaderm to minimize mechanical stress on the wounds and surrounding skin. All applications were performed by trained personnel under supervision, following dressing removal, safety assessment, and exploratory sampling.

Details of dose levels are provided in Table 1, which were based on the effective doses identified in vitro and in vivo [18, 33] and the rationale for these doses is further elaborated in the Supporting Information.

**TABLE 1:** Dose levels.

Dose group (mg/mL)	Wound size (cm^2^)	Volume/wound (mL)	Dose/cm^2^ wound (mg/cm^2^)	Dose/wound (mg)	Total daily dose in 2 wounds (mg)
TCP‐25 0.86 mg/mL or placebo	0.8	0.15	0.16	0.13	0.26
TCP‐25 2.9 mg/mL or placebo	0.8	0.15	0.54	0.44	0.87
TCP‐25 8.6 mg/mL or placebo	0.8	0.15	1.61	1.29	2.58

Dose escalation occurred following review by an internal Safety Review Committee based on safety, tolerability, and pharmacokinetic data from the previous dose group, as detailed in the Supporting Information and Table S1. Safety evaluations and stopping criteria adhered to Common Terminology Criteria for Adverse Events (CTCAE) v5.0 guidelines and those of Sibille et al. [34] and are further detailed in the Supporting Information.

### 2.5 Primary Endpoints

The primary aim was to collect data on the frequency, intensity, and seriousness of AEs, including local tolerability, and to evaluate any clinically significant changes from baseline in electrocardiogram (ECG), vital signs, and laboratory parameters.

AEs, including serious adverse events (SAEs) that were reported by the participant, observed by the investigator or medical personnel, or elicited by nonleading questions from the investigator or medical personnel were recorded at each study visit from the start of gel administration until the end‐of‐study visit.

AEs included local tolerability parameters (as defined in the Supporting Information), abnormal findings with regard to ECG, vital signs, and safety laboratory parameters (as defined in the Supporting Information), infections, and unexpected and excessive hemorrhage. Coagulated blood in wounds was not considered an AE.

AEs were classified by the investigator into categories, and their severity was graded from 1 to 5 according to the common terminology criteria in CTCAE v5.0 [35]. AEs were also assessed by the investigator as unlikely, possibly, or probably related to the treatment, according to the definitions detailed in the Supporting Information.

### 2.6 Secondary Endpoints

The secondary aim was to evaluate systemic exposure to TCP‐25 after topical application. Venous blood (approximately 5 mL) was collected by venipuncture or an indwelling venous catheter. Plasma TCP‐25 concentrations were measured at the following timepoints:

- Day 1: prior to wound creation
- Day 2: predose and 30 min and 1 h after administration of the gel
- Day 3: predose and 1 h postadministration
- Day 5: predose only

The plasma separated from the blood sample was frozen at < −70°C within 1 h after centrifugation. The samples were analyzed by Q&Q Laboratories AB (Gothenburg, Sweden) using a validated liquid chromatography–tandem mass spectrometry method. The lower limit of quantification was 90 nmol/L.

### 2.7 Exploratory Endpoints

Photographs of wounds with and without primary dressings were taken using the method described in detail in Wallblom et al. [36]. Briefly, a ruler was placed near wounds, and wound/dressing images were captured at 35 cm with a Canfield Twin Flash camera and a modified Canfield's close‐up scale (Canfield Scientific, Parsippany‐Troy Hills, USA). Dressing images were taken using a polarizing filter (Canfield, USA) to remove reflections and visualize the Mepilex dressing beneath the transparent Tegaderm wound film.

Wound exudation was quantified gravimetrically by weighing used dressings. At each dressing change, the standardized 2 × 2‐cm Mepilex dressings were removed and placed into 5‐mL tubes. Baseline weights were established using six unused dressings and six empty tubes (mean combined weight: 3285.4 mg). The net exudate weight was calculated by subtracting the baseline weight from the total weight of each sample. This method provided a semi‐quantitative measure of exudation [37] recognizing that the semi‐permeable dressing material (Mepilex and Tegaderm) permits moisture evaporation and may underestimate actual fluid output.

Dressing yellowness, a surrogate for leakage, was quantified in dressing images using ImageJ (version 1.54p, National Institutes of Health, USA) by converting RGB to CIELAB and extracting the b* value (yellow intensity), as described in Wallblom et al. [36]. The dressing area was selected with ImageJ's polygonal tool, and the mean b* value was calculated. The assessor was not blinded during the yellowness analysis. As a complementary manual visual assessment of dressing images, 3 independent physicians compared each pair of wounds to determine which wound (TCP‐25‐treated, placebo‐treated, or neither [no discernible difference]) exhibited the least visual signs of wound exudation. For this analysis, dressing images were randomized, and assessors were blinded to treatment allocation, imaging timepoint, dose group, and participant identity by masking this information in images; however, the wound location (L1, L2, R1, and R2) remained visible in images.

The open wound area was quantified in wound images using digital planimetry in ImageJ similarly as done in Larsen et al. [31]. Briefly, the image scale was calibrated by measuring 1 cm on the ruler visible in each photograph. The wound margins were then manually traced, and the open wound area (in cm^2^) was calculated by ImageJ.

Extraction of wound fluid was performed as previously described by Lundgren et al. [25]. Briefly, dressings collected on Days 2, 3, 5, 8, and 11 were extracted with cold Tris buffer, and the fluids were supplemented with protease inhibitors before aliquoting and storage at −80°C. Protein concentration was determined using the bicinchoninic acid (BCA) assay (Thermo Fisher, Waltham, MA, USA) with bovine serum albumin as the standard. The concentration in mg/mL was calculated from the standard curve, and total protein content per dressing was obtained by multiplying this value by the total extract volume [25].

### 2.8 Statistics

#### 2.8.1 Primary and Secondary Outcomes

Continuous data are presented in terms of evaluable observations, arithmetic mean, standard deviation (SD), median, minimum, and maximum value. Categorical data are presented as counts and percentages. When applicable, summary data are presented by treatment and assessment time. Individual participant data are listed by participant number, treatment, and, where applicable, assessment time. Baseline was defined as the visit with the last data collection point prior to the first administration of treatment. All descriptive summaries and non‐exploratory statistical analyses were performed using SAS, version 9.4 or later (SAS Institute Inc., Cary, NC, USA). Generally, no imputation of data was performed. Laboratory parameters outside the limits of detection were replaced with the detection limit for statistical calculations. We analyzed all data for the intention‐to‐treat population, which comprised all randomized participants. Statisticians were not blinded for any analyses. An excerpt from the clinical study plan, covering the relevant statistical sections, is included in the Supporting Information.

#### 2.8.2 Exploratory Analyses

Inter‐rater agreement for visual assessment of wound exudation by the 3 scorers was evaluated using quadratic‐weighted Fleiss kappa (irrCAC package version 1.0, R version 4.5.2) [38]. The strength of agreement was evaluated according to the guidelines by Landis et al. [39].

Correlation analyses were performed between total protein content and clinical exudation metrics (visual assessment and gravimetric measurement) to investigate the relationship between visual appearance and both protein leakage and exudate weight. Correlation between the median visual assessment of 3 physicians and the difference in quantitative data (CIELAB b*, total protein content, and exudate weight) was analyzed by calculating Spearman's correlation coefficient. Analysis was done both for pooled data from all timepoints and for data separated by timepoint. The strength of the correlation was evaluated according to the guidelines by Schober et al. [40].

Comparisons between placebo and TCP‐25‐treated wounds for yellowness (CIELAB b*), exudate weight, and open wound size were performed using Wilcoxon matched‐pairs signedrank tests at each timepoint separately. For each participant, values from the two wounds per treatment group were averaged prior to analysis. No adjustments for multiple comparisons were applied, as these analyses were exploratory. GraphPad Prism version 10.4.1 was used for data visualization and analysis.

Data supporting the exploratory analyses are provided in the Supporting Information file. Other data supporting the findings of this study are available from the corresponding author upon reasonable request. Certain data availability is subject to privacy restrictions to protect the participants' privacy.

## 3 Results

### 3.1 Participant Flow

**FIGURE 2:**
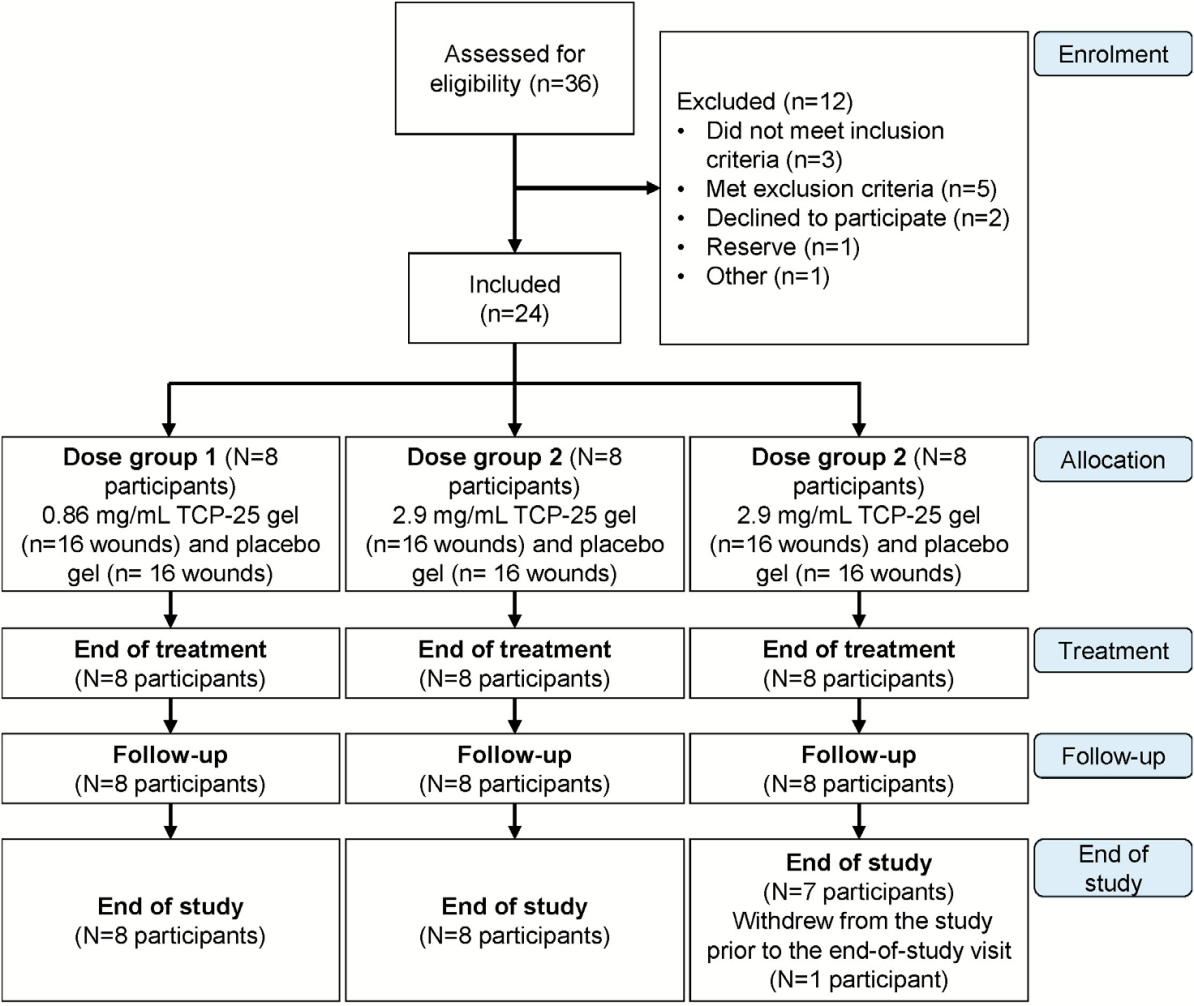
Participant flow diagram. Multiple topical doses of TCP‐25 were administered in 3 sequential dose groups (*n* = 8). An internal safety review committee reviewed the safety, tolerability, and pharmacokinetic data prior to each dose escalation. One participant withdrew from the study and did not attend the end‐of‐study visit.

In total, 36 healthy volunteers were screened and 24 were randomized (as planned). All randomized participants received 2 acute epidermal wounds on each thigh, formed using the suction blister technique, yielding a total of 96 wounds. The participant flow is shown in Figure 2. The first participant was screened on April 7, 2022 and randomized on April 25, 2022. The last visit of the last participant occurred on June 20, 2022.

Eight participants were screening failures, 2 eligible participants withdrew consent before randomization, 1 eligible participant was not needed in the study (becoming a reserve participant), and 1 participant was initially non‐eligible but was rescreened and found to be eligible.

All participants received 5 topical dose applications of TCP‐25 or placebo gel over 8 days, resulting in 100% treatment compliance. One participant in the 8.6‐mg/mL group withdrew from the study for personal reasons after completing the follow‐up visit on Day 11 and hence did not attend the end‐of‐study visit.

### 3.2 Participant Baseline Characteristics and Demographics

The included participants comprised 16 females (67%) and 8 males (33%), and the median age was 22.5 years (range, 18–57 years). Overall, the demographics and baseline characteristics were comparable between the dose groups, except that all participants in the 2.9‐mg/mL group were female. The demographic and baseline data of all randomized participants are presented in Table 2.

**TABLE 2:** Participant characteristics at baseline.

Assessment	TCP‐25 0.86 mg/mL (*N* = 8)	TCP‐25 2.9 mg/mL (*N* = 8)	TCP‐25 8.6 mg/mL (*N* = 8)	All (*N* = 24)
Age (years); median (range)	20.5 (19.0–42.0)	24.5 (20.0–30.0)	23.5 (18.0–57.0)	22.5 (18.0–57.0)
Height (cm); median (range)	172.0 (159.0–188.0)	165.5 (161.0–180.0)	183.0 (168.0–196.0)	172.0 (159.0–196.0)
Weight (kg); median (range)	66 (47–86)	65 (50–78)	73 (54–91)	68 (47–91)
Females; *n* (%)	5 (63%)	8 (100%)	3 (38%)	16 (67%)
Ethnicity; *n* (%)				
Not Hispanic or Latino	8 (100%)	6 (75%)	8 (100%)	22 (92%)
Not reported	0 (0%)	2 (25%)	0 (0%)	2 (8%)
Race				
Asian	1 (13%)			1 (4.2%)
White	7 (88%)	8 (100%)	8 (100%)	23 (96%)

Fifteen of 24 participants (63%) reported medical history, most commonly psychiatric disorders (25%) and surgical and medical procedures (17%). No clinically relevant group differences were observed.

### 3.3 AEs and Local Tolerability

A total of 21 participants (88%; 7 of 8 participants in each dose group) reported at least 1 AE (Tables S2–S5). No deaths, SAEs, other significant AEs, or withdrawals due to AEs were observed. All 61 reported AEs were assessed as unlikely to be related to the treatment, and all were assessed as mild (46 events) or moderate (15 events) in intensity. There was no apparent dose dependency of the number of AEs, the types of AEs that were reported, or AE intensity. No difference in wound‐associated AEs was observed between wounds treated with TCP‐25 or placebo gel.

The most common AEs were skin irritation (21 events reported by 14 participants) and folliculitis (6 events in 5 participants). All events of skin irritation and folliculitis were judged to be associated with the overlying dressing, film, or tape.

There were no abnormal local reactions (erythema, edema, necrosis, crusting, and hemorrhage or purulent discharge) compared with expected wound healing outcomes in any TCP‐25 or placebo‐treated wound at any time.

### 3.4 Concomitant Medication

Concomitant medications were used by 21 participants (88%), most commonly topical corticosteroids (betamethasone valerate, 11 participants, 46%). Topical corticosteroids were administered to treat 13 AEs of skin irritation and erythema (12 mild, 1 moderate), all attributed to the adhesive tape and occlusive film and judged by the investigators to be unlikely to be related to TCP‐25 treatment. These events occurred at a median of Day 5 (range 2–15) with a median duration of 8 days (range 4–11). At the end‐of‐study visit, 8 of these AEs had resolved, 4 were resolving, and 1 had not yet resolved. Topical corticosteroid use was distributed across dose groups: 5, 5, and 1 participants in the 0.86‐, 2.9‐, and 8.6‐mg/mL groups, respectively. Topical corticosteroids were applied only to intact affected skin, away from the wound and the treated area beneath the Mepilex transfer dressing, and the investigators judged their use unlikely to interfere with the wound area, wound healing, or the study's objectives. The second most common concomitant medication was paracetamol (6 participants, 25%), most commonly used due to headache (3 participants).

Other than topical corticosteroids for skin irritation, there were no major differences in the use of prior or concomitant medications or the reason for the use of concomitant medications between the dose groups. There was no indication of increased use of concomitant medications with escalating doses of TCP‐25. No use of prior or concomitant medication was judged by the investigator to have interfered with the evaluation of the study endpoints.

### 3.5 Safety Measurements

There were no clinically relevant changes in ECG parameters, vital signs, or physical examination findings from baseline to the end of treatment on Day 11 in any dose group. No indications of dose‐dependent effects on any safety parameters were observed.

One participant had hemoglobin levels slightly below the reference value on Day 11 and was judged to be mildly anemic. This finding was reported as an AE that was assessed as being unlikely to be related to treatment. Another participant had low thrombocyte levels on Day 11, which was also reported as an AE (thrombocytopenia) but assessed as unrelated to the treatment.

### 3.6 Systemic Exposure to TCP‐25

TCP‐25 levels were below the lower limit of quantification (90 nmol/L) in plasma samples from all participants at all timepoints. Consequently, no further analyses of pharmacokinetic parameters could be performed.

### 3.7 Exploratory Analysis

**FIGURE 3:**
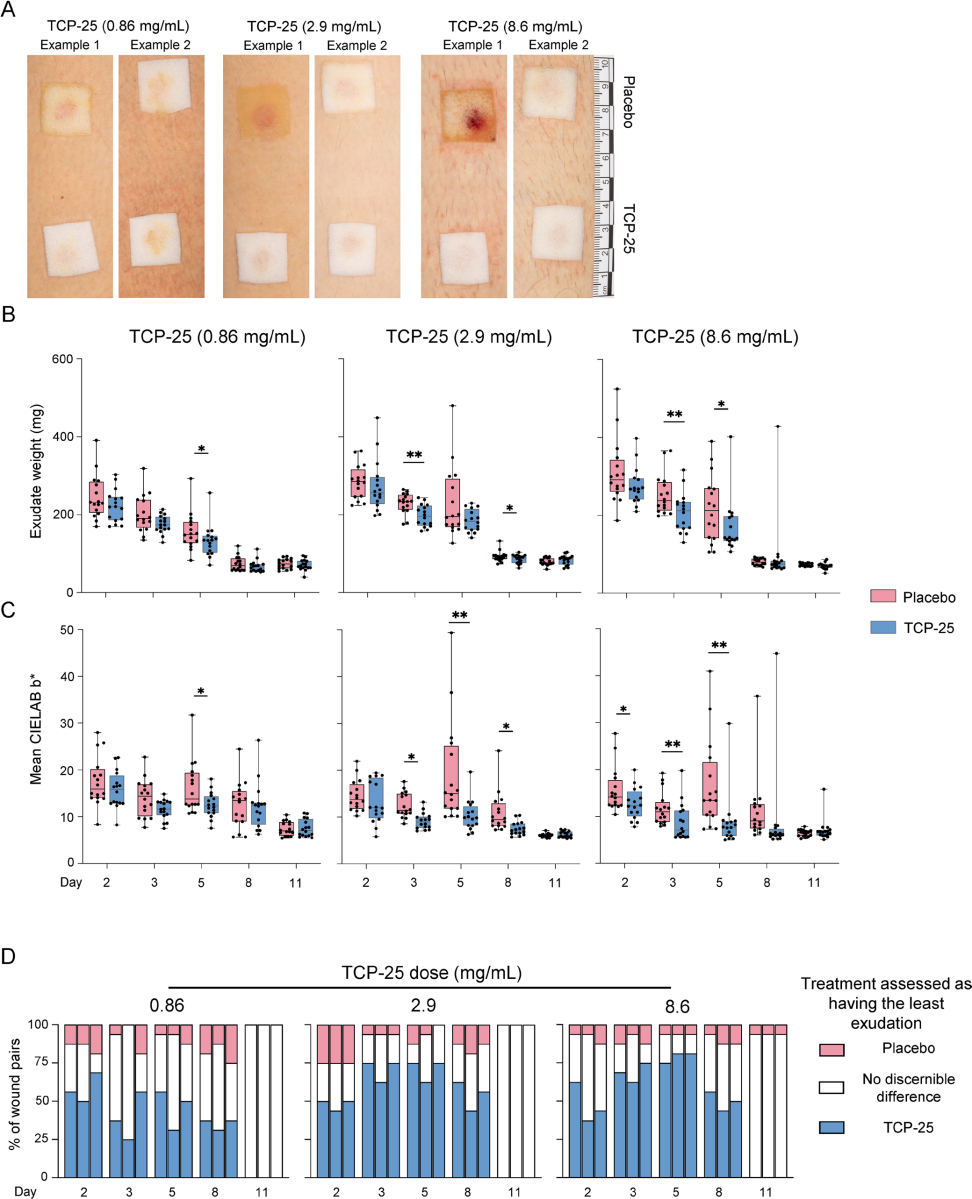
Exploratory analyses indicate a decrease in exudation with TCP‐25 treatment. (A) Representative dressing photographs from Day 5. Two examples from each TCP‐25 dose group illustrate the maximum observed difference (Example 1) and the median difference (Example 2) in dressing appearance between TCP‐25 and placebo treatments. (B, C) Box plots depict median (center line), interquartile range (box), and range (whiskers); points represent individual wounds (32 wounds from 8 participants per dose group and timepoint). For statistics, values from the two wounds per treatment within each participant were averaged, yielding *n* = 8 paired observations per dose group and timepoint. Comparisons between TCP‐25 and placebo at each timepoint used the Wilcoxon matched‐pairs signed–rank test. No multiple comparison adjustments were applied, as the analysis was exploratory in nature. *p < 0.05, **p < 0.01 (B) Exudate weight over time. TCP‐25 treatment reduced exudate weight compared with placebo on Days 3 (2.9 and 8.6 mg/mL groups) and 5 (0.86 and 8.6 mg/mL groups). (C) Dressing yellowness (CIELAB b*) over time. TCP‐25‐treated wounds demonstrated reduced dressing yellowness compared with placebo, particularly on Day 5 across all dose groups. (D) Manual visual assessment over time. Three physicians, blinded to treatment, independently reviewed paired dressing images (TCP‐25 vs. placebo within participant) and indicated which corresponding dressing showed the least visible signs of exudation (TCP‐25, placebo, or no discernible difference). For each dose × day, three stacked bars are shown (one per physician; left‐to‐right order Assessor 1–3), displaying the percentage of wound pairs assigned to each category. *n* = 16 wound pairs per dose group and timepoint (8 participants; 2 pairs/participant).

Representative dressing images from each dose group on Day 5 illustrate both maximal and median observed differences between placebo and TCP‐25 treatment (Figure 3A).

Exudate weight is an objective and semi‐quantitative measure of wound exudation (Figure 3B). The exudate weight was generally highest on Day 2 and gradually decreased until Day 11, when low exudate weights were recorded for both TCP‐25 and placebo‐treated wounds. Differences in exudate weight indicating reduced exudation with TCP‐25 treatment were observed on Day 3 (2.9 and 8.6 mg/mL: *p* = 0.008 each) and Day 5 (0.86 mg/mL: *p* = 0.04; 8.6 mg/mL: *p* = 0.02). Analysis of individual wound pairs over time, visualized in heat maps (Figure S1), demonstrated a consistent reduction in exudate weight with TCP‐25 treatment. Greater reductions in exudate weight were generally observed with higher TCP‐25 concentrations (2.9 and 8.6 mg/mL) compared with 0.86 mg/mL.

To quantify visual differences, we analyzed dressing yellowness (CIELAB b*) as a marker of exudation [36]. Peak discoloration generally occurred earlier in TCP‐25‐treated wounds (Day 2) compared to placebo‐treated wounds (Day 5) (Figure 3C). By Day 11, discoloration was minimal across all treatment groups. The greatest treatment differences were observed on Day 5, when all TCP‐25 concentrations reduced dressing yellowness compared to placebo (0.86 mg/mL: *p* = 0.02; 2.9 mg/mL: *p* = 0.008; 8.6 mg/mL: *p* = 0.008). To visualize treatment differences in individual wound pairs over time, heat maps illustrating the difference in dressing yellowness for each wound pair are included in Figure S1. Notably, a greater reduction in dressing yellowness was generally observed with higher TCP‐25 concentrations (2.9 and 8.6 mg/mL) compared to the 0.86 mg/mL concentration.

As a complementary visual assessment of exudation, three physicians blinded to treatment allocation independently reviewed paired dressing photographs (TCP‐25 vs. placebo within participant) and indicated which treatment showed the least visible evidence of exudation (TCP‐25, placebo, or no discernible difference). Figure 3D presents the distribution of classifications for each assessor separately (three bars per dose × day). Across assessors, TCP‐25 was more frequently selected as having less visible exudation than placebo at all doses and timepoints except Day 11 (Figure 3D). By Day 11, nearly all wound pairs were scored as showing no discernible difference, with a single pair at 8.6 mg/mL scored in favor of placebo. Overall, the visual scoring trends were consistent with the computer‐assisted measures. Inter‐rater agreement was substantial between the 3 assessors (quadratic‐weighted Fleiss' kappa = 0.842, 95% CI 0.799–0.885). Correlation analyses examined relationships between the visual assessment and the difference (TCP‐25‐treated wound—placebo‐treated wound) in quantitative measures: dressing yellowness (CIELAB b*), exudate weight, and total dressing protein content. Spearman's correlation analysis on pooled data across all dose groups and timepoints indicated a strong correlation of physician assessment of exudation with dressing yellowness (*r* = 0.84) and total protein content (*r* = 0.72), and a moderate correlation with exudate weight (*r* = 0.57) (Figure S2). Timepoint‐specific sensitivity analysis (excluding Day 11, as all pairs but one were assessed as equal) yielded similar results (Figure S2).

Planimetric analysis of digital wound images showed comparable wound size at Day 1 prior to treatment (placebo: mean 0.80 cm^2^, SD 0.01; TCP‐25: mean 0.79 cm^2^, SD 0.09). Open wound area decreased over time in both groups, with the majority of wounds closed by Day 8 and all closed by Day 11 (Figure S3). No statistically significant differences in open wound size were detected between TCP‐25 and placebo‐treated wounds at any dose or timepoint (all *p* > 0.18).

## 4 Discussion

This first‐in‐human, Phase 1 study demonstrated that topical TCP‐25 gel was safe and well‐tolerated when applied to epidermal suction blister wounds in 24 healthy volunteers. All recorded AEs were mild to moderate, with no clinically relevant differences between TCP‐25‐treated and placebo‐treated wounds. Common AEs, such as skin irritation and folliculitis, were attributed to the dressings or adhesive tape rather than the gel [41]. While these dressing‐related skin reactions were confined to the surrounding intact skin outside the wound evaluation area and were judged not to influence study outcomes, future iterations of this wound model could incorporate dressings with lower irritancy potential and reduce surface coverage to further minimize such events. Topical betamethasone, administered to manage these adhesive‐related skin reactions at a distance from the wounds, was judged unlikely to influence wound healing or study outcomes given the spatial separation from the wound area, limited lateral diffusion and systemic absorption of topical corticosteroids at the doses used [42, 43] and the within‐participant paired‐wound design ensuring that any potential effects would equally affect both TCP‐25 and placebo wounds.

No abnormal local reactions or deviations from expected wound healing were observed, indicating that topical TCP‐25 does not interfere with normal physiological wound repair in healthy individuals. All laboratory parameters, ECG, vital signs, and physical examination findings remained within normal limits, except for 2 isolated out‐of‐range laboratory values, classified as unrelated to TCP‐25. These safety findings are consistent with previous preclinical studies in murine and porcine models [18, 33] and align with the biological rationale that the natural presence of thrombin and endogenous TCP sequences in human wound fluid minimizes the potential for adverse immunological reactions [16, 17].

TCP‐25 was undetectable in plasma samples from all participants at all timepoints, confirming the absence of detectable systemic absorption following topical application. The results are consistent with preclinical findings demonstrating no uptake of TCP‐25 at 2 and 24 h after topical application to porcine skin and larger partial‐thickness wounds [18]. TCP‐25 is a positively charged peptide with a molecular weight above 3 kDa, and charged peptides above 1 kDa typically exhibit poor penetration across the skin barrier [44, 45]. Even in open wounds where the epidermal barrier is compromised, fewer than 1% of applied peptides are expected to achieve systemic uptake [46]. Thus, the lack of any detectable systemic uptake of TCP‐25 in our study aligns with its expected behavior based on preclinical studies and its molecular properties.

Regarding the local bioavailability of TCP‐25, previous studies have shown that TCP‐25 exhibits minimal binding to both the neutral hydrogel matrix [18] and to polyurethane‐based Mepilex dressings [33], with rapid and efficient peptide release and preserved biological activity in both systems. These findings indicate that the dressing materials used in this study do not sequester TCP‐25 or restrict its availability at the wound surface.

The exudate‐reducing effect of TCP‐25 observed in this study has not been described previously but may hold clinical relevance. While physiological exudation is essential for normal wound healing, excessive exudate complicates wound management by necessitating frequent dressing changes, causing skin maceration, and creating a favorable environment for microbes [37]. Complex wounds, such as those in EB and venous ulcers in elderly patients, are particularly challenging due to a combination of persistent inflammation, bacterial colonization, and high exudate levels [1, 12, 14, 37]. Current treatment options, including silver‐containing dressings and antiseptics, only target wound bacteria but do not directly address the underlying excessive inflammation and exudation [47]. In this context, the observed exudate‐reducing effect of TCP‐25, combined with its well‐characterized anti‐inflammatory and antibacterial properties [18, 19, 20, 23, 48] could represent a novel treatment approach for complex wounds. However, the clinical translation of these effects to complex human wounds remains to be established. Furthermore, since vascular permeability is a key mediator of exudation [37, 49] but was not evaluated in this study, a mechanistic investigation of related pathways is warranted.

The methodology used adds significant strength to the clinical trial. The suction blister technique provided a standardized, reproducible model of acute epidermal wounds that approximates the target indication of EB wounds. Unlike punch biopsies, suction blister wounds heal without scarring, rendering them suitable for safety and tolerability studies. The study employed a sequential three‐dose cohort design, with an internal safety review committee evaluating emerging safety data before each dose escalation to ensure safety. The randomized, blinded, placebo‐controlled design, in addition to the use of internal controls (wherein each participant served as their own control), minimized variability and enhanced reliability.

Several limitations warrant consideration. This study involved healthy volunteers without underlying conditions affecting wound healing. Epidermal suction blister wounds lack the complexity of disease‐induced wounds, as they typically heal in a highly predictable manner without complications such as scarring. The limited wound size and short treatment duration also do not reflect the clinical context of hard‐to‐heal wounds, which are often larger and require prolonged treatment. In addition, the study was conducted as a single‐center study with 24 participants of predominantly Caucasian origin, limiting generalizability. While these constraints were appropriate for a first‐in‐human safety and tolerability study, they limit the ability to extrapolate results to larger, more complex wounds.

## 5 Conclusion

In this study—the first in a 3‐part Phase 1 clinical trial—TCP‐25 gel, administered topically at 3 strengths (0.86, 2.9, and 8.6 mg/mL), was safe and well tolerated by healthy volunteers in whom experimental wounds were induced by the suction blister technique. No systemic uptake of TCP‐25 was detected. These results provided the basis for subsequent clinical safety evaluation in chronic leg ulcers (Part 2) and, as recently reported, in wounds of patients with EB (Part 3) [50].

## Author Contributions

K.W., J.F., A.S., and M.H. wrote the manuscript; K.W., S.L., G.P., M.P., K.S., A.S., and M.H. designed the research; K.W., S.L., G.P., M.P., J.F., and K.S. performed the research; K.W., J.F., and A.S. analyzed the data.

## Funding

This safety study was funded by Xinnate AB. Exploratory analyses on data and materials originating from this study are integrated in this and other research projects funded by research grants from the Swedish Research Council (projects 2020‐02016 and 2025‐02401), Edvard Welanders Stiftelse and Finsenstiftelsen (Hudfonden), the Österlund Foundation, and the Swedish Government Funds for Clinical Research (ALF).

## Conflicts of Interest

A.S. is a founder of and holds shares in in2cure AB, a parent company of Xinnate AB, which was the sponsor of the study and holds patents for TCP‐25. G.P. was employed part‐time (20%) by Xinnate AB during the study. M.H. is employed by Xinnate AB. The other authors declared no competing interests for this work.

## Supporting information


**Data S1:** cts70497‐sup‐0001‐supinfo.xlsx.

**Data S2:** cts70497‐sup‐0002‐supinfo.docx.

**Data S3:** cts70497‐sup‐0003‐supinfo.pdf.
