# Supplementary material for: A First‐In‐Human Randomized Controlled Phase 1 Study Assessing the Safety and Tolerability of Topical TCP‐25 Gel in Epidermal Suction Blister Wounds
Source: Clin Transl Sci. 2026 Feb 5;19(2):e70497. doi: 10.1111/cts.70497 (PMC12877310; doi:10.1111/cts.70497)
Supplement: Supplementary file 2 — Data S2: cts70497‐sup‐0002‐supinfo.docx. [file CTS-19-e70497-s003.docx]

**A First-in-Human Randomized Controlled Phase 1 Study Assessing the Safety and Tolerability of Topical TCP-25 gel in Epidermal Suction Blister Wounds**

Contents

[Inclusion criteria 2](#_Toc218509665)

[Exclusion criteria 3](#_Toc218509666)

[Study withdrawal 4](#_Toc218509667)

[Baseline characteristics 5](#_Toc218509668)

[Data monitoring 5](#_Toc218509669)

[Randomization and blinding 6](#_Toc218509670)

[Dose rationale 6](#_Toc218509671)

[Internal safety review committee, dose escalation, and stopping criteria 7](#_Toc218509672)

[Table S1 7](#_Toc218509673)

[Local tolerability 9](#_Toc218509674)

[ECG, vital signs, and safety laboratory parameters 9](#_Toc218509675)

[Definition of causality of adverse events 10](#_Toc218509676)

[Adverse events 11](#_Toc218509677)

[Table S2 11](#_Toc218509678)

[Table S3. 12](#_Toc218509679)

[Table S4. 14](#_Toc218509680)

[Table S5 18](#_Toc218509681)

[Exploratory analysis 20](#_Toc218509682)

[Figure S1 20](#_Toc218509683)

[Figure S2 22](#_Toc218509684)

[Figure S3 22](#_Toc218509685)

[References 23](#_Toc218509686)

# Inclusion criteria

For inclusion in the study, participants had to fulfil the following criteria:

1. Willing and able to give written informed consent for participation in the study.
2. Healthy male or female participant 18-60 years (inclusive) of age at the time of signing the informed consent.
3. Body Mass Index (BMI) ≥ 18.0 and ≤ 30.0 kg/m^2^.
4. Healthy and intact skin where the blister suction wounds were to be induced.
5. Women of childbearing potential (WOCBP) had to have a documented negative serum pregnancy test done at the screening visit, within 4 weeks prior to suction blister formation and the start of study treatment.
   1. WOCBP had to practice abstinence (only allowed when this was the preferred and usual lifestyle of the participant) or had to agree to use a highly effective method of contraception with a failure rate of < 1% to prevent pregnancy (combined [oestrogen and progestogen containing] hormonal contraception associated with inhibition of ovulation [oral, intravaginal, transdermal], progestogen-only hormonal contraception associated with inhibition of ovulation [oral, injectable, implantable], intrauterine device or intrauterine hormone-releasing system) from at least 4 weeks prior to dose to 4 weeks after last dose. Female participants had to refrain from donating eggs from the date of dosing until 3 months after dosing with the IMP. Their male partner had to agree to use a condom during the same time frame if he had not undergone vasectomy.
   2. Women of non-childbearing potential were defined as pre-menopausal females who were sterilised (tubal ligation or permanent bilateral occlusion of fallopian tubes); or females who had undergone hysterectomy or bilateral oophorectomy; or post-menopausal defined as 12 months of amenorrhea (in questionable cases a blood sample with detection of follicle stimulating hormone 25-140 IU/L was confirmatory).
   3. Male participants had to be willing to use condom or be vasectomised or practice sexual abstinence to prevent pregnancy and drug exposure of a partner and refrain from donating sperm from the date of the last dosing until 3 months after the last dosing with the IMP. Their female partner of childbearing potential had to use contraceptive methods with a failure rate of < 1% to prevent pregnancy (see above).
6. Clinically relevant medical history, physical findings, vital signs, ECG, and laboratory values at the time of screening, as judged by the Investigator.

# Exclusion criteria

Participants could not enter the study if any of the following exclusion criteria were fulfilled:

1. History of any clinically significant disease or disorder which, in the opinion of the Investigator, could either put the participant at risk because of participation in the study, or influence the results or the participant’s ability to participate in the study.
2. Disease that could interfere with wound healing, *e.g.,* diabetes type I/II, arterial-, renal-, liver, or cardiac insufficiency, chronic obstructive lung disease, cancer, autoimmune disease, oedema at the study site, severe obesity, or previous known wound healing problems, as judged by the Investigator.
3. Active skin disease, *e.g.,* dermatitis, psoriasis, wounds, and/or tattoos in the areas where suction blister wounds were to be induced, as judged by the Investigator.
4. Any planned major surgery within the duration of the study.
5. After 10 minutes supine rest at the time of screening, any vital signs values outside the following ranges:
   1. Systolic blood pressure <90 or >160 mmHg, or
   2. Diastolic blood pressure <50 or >100 mmHg, or
   3. Pulse <40 or >90 beats per minute (bpm)
6. Any clinically significant abnormalities in the resting ECG at the time of screening, as judged by the Investigator.
7. Current smokers or users of nicotine products. Irregular use of nicotine (*e.g.,* smoking, snuffing, chewing tobacco) less than 3 times per week was allowed before the screening visit.
8. Female participants who were pregnant or lactating or planned a pregnancy.
9. Systemic immunosuppressive treatment.
10. Participants receiving or had received the following treatments within 2 weeks prior to screening:
    1. systemic corticosteroids or immunosuppressant agents; or
    2. antibiotics via any route
11. Regular use of anticoagulants (*i.e.,* heparin, warfarin, coumarins, other anticoagulants per Investigator’s judgement) or non-steroidal anti-inflammatory drugs (NSAIDs) within 2 weeks prior to the (first) administration of IMP, at the discretion of the Investigator.
12. History of severe allergy/hypersensitivity or ongoing allergy/hypersensitivity, as judged by the Investigator, or history of hypersensitivity to drugs with a similar chemical structure or class to TCP-25 or to any excipients of the hydrogel.
13. Planned treatment or treatment with another investigational drug within 3 months prior to Day -1.
14. History of alcohol abuse or excessive intake of alcohol, as judged by the Investigator.
15. Presence or history of drug abuse, as judged by the Investigator.
16. Plasma donation within 1 month of screening or blood donation (or corresponding blood loss) during the 3 months prior to screening.
17. Involvement in the planning and/or conduct of the study.
18. Investigator considered the participant unlikely to comply with study procedures, restrictions, and requirements.

# Study withdrawal

Participants were free to discontinue study participation at any time without affecting their right to appropriate follow-up investigation or future care. When possible, the reason for withdrawal of consent was documented.

Participants could be discontinued from the study at any time at the investigator's discretion for the following reasons:

- Participant decision to withdraw
- Severe non-compliance with study protocol procedures, as determined by the investigator and/or sponsor
- Loss to follow-up
- Significant adverse events posing risk to the participant, as determined by the investigator and/or sponsor
- Withdrawal of informed consent for use of biological samples
- Pregnancy
- Death
- Development of exclusion criteria during the study that, in the investigator's opinion, posed risk to the participant
- Use of prohibited medication

Participants who prematurely discontinued study participation were questioned regarding reason(s) for discontinuation and presence of any adverse events. When participants withdrew consent, investigators asked whether they were willing to undergo end-of-study visit procedures as soon as possible. Ongoing adverse events were followed according to standard procedures. The primary reason for discontinuation or early withdrawal was documented for each case.

# Baseline characteristics

The following demographic and baseline data were recorded in the eCRF: sex, age, ethnicity, race, weight, and height. Medical/surgical history and prior medications were obtained by interviewing the participant and included all relevant diseases and surgeries within 2 months prior to the screening, as judged by the investigator. All concomitant medications were registered. Medications were classified as “prior” if the stop date was before or on the day of administration of the first dose (predose) and as “concomitant” if their use was ongoing on the day that the first dose was administered or if they were stopped or started after the first dose had been administered. Any use of concomitant medication from the screening until the last end-of-study visit was documented. Medical/surgical history was coded using the Medical Dictionary for Regulatory Activities (MedDRA), version 24.1. Medications were coded using the WHO Drug Dictionary (WHODD) 2021.

# Data monitoring

Data were captured by the investigator in an electronic case report form (eCRF) (Viedoc Technologies AB, Uppsala, Sweden). The study was monitored to ensure that the participants’ rights, safety, and well-being were met and that the data in the CRF were complete, correct, and consistent with the source data. Data management was performed by CTC in accordance with the data management plan (DMP). When all data had been cleaned, coded, validated, signed, and locked, clean file was declared, and the database for each part of the trial was locked.

# Randomization and blinding

The randomization list was read by an unblinded research nurse in a nearby medical room to determine the assigned allocation of TCP-25 and control gel for each participant. The research nurse then filled 2 syringes per participant: 1 containing TCP-25 gel and the other containing placebo gel (produced as described below). The syringes were labeled only with the randomization number of the participant and the assigned wounds—"R1+L2” or “R2+L1”—that were to receive the contents of that syringe per the treatment allocation. These prefilled and labeled syringes were provided to blinded research staff, who then applied their contents onto the wounds as indicated on the syringe label.

Sealed individual treatment code envelopes were stored at the clinic in a locked and restricted area and in CTC’s Pharmacovigilance Department if needed for emergency unblinding.

# Dose rationale

The dose range and dosing schedule for TCP-25 were selected based on microbiological, pharmacological, and toxicological data from the preclinical program. TCP-25 demonstrated MICs against Staphylococcus aureus of 7.7–30.9 µg/mL (one isolate: 247.1 µg/mL), against Pseudomonas aeruginosa of 30.9–247.1 µg/mL (one isolate: 0.49 mg/mL), and against Escherichia coli of 3.7–30.9 µg/mL (1), with similar values obtained for several multi-resistant clinical isolates (2). Based on these results, a concentration range of 0.86 to 8.6 mg/ml TCP-25, corresponding to approx. 0.28 to 2.8 mM was chosen to ensure bactericidal activity at the wound surface, even with dilution by wound exudate.

This concentration range also exceeds the levels required for CD14 and LPS binding (≥ 2 µM) (3), consistent with the observed significant anti-inflammatory effect in vivo, which was further supported by analyses of TCP-25 concentration in wound fluid (2) and *in vitro* studies using macrophages and human blood (4).

The 24-hour dosing interval used in the present study mirrors the regimen shown to be effective in the porcine model, where daily applications initially, followed by reduced frequency as exudation decreased, resulted in significant reductions in inflammation and bacterial burden (2).

In preceding studies TCP-25 showed no measurable systemic absorption even when administered to substantially larger and deeper wounds: both the porcine partial thickness wound model (2) and repeat-dose toxicity studies in minipigs using full-thickness wounds (35 mg/day TCP-25 for 28 days) (5) yielded plasma levels below the limit of quantification. Showing that TCP-25 remains locally confined with no measurable systemic uptake.

Traditional allometric scaling could therefore not be applied systemically, as TCP-25 shows no measurable systemic absorption even when administered to substantially larger and deeper wounds. However, conservative human-equivalent dose calculations, assuming 100% uptake of topically applied TCP-25, based on no observed adverse effect level (NOAEL) values from both mice and minipigs yielded substantial safety margins, 56-fold relative to the mouse NOAEL and 450-fold relative to the minipig NOAEL (5).

# Internal safety review committee, dose escalation, and stopping criteria

The voting members of the internal safety review committee (iSRC) consisted of the PI or delegate and the Co-Investigator or delegate. In addition, the study clinical research manager, the study pharmacokineticist, additional Sponsor representatives and/or other experts were invited as appropriate. Dose escalation required iSRC approval following complete treatment and safety evaluation of all participants in the preceding dose group. Safety and tolerability data (at least 8 days) and pharmacokinetic data (at least 5 days) were reviewed for all participants before advancing to the next dose level.

Table S1**.** Dose escalation and termination rules for individual and dose group level

| **Stop criterion** | **Action taken** |
| --- | --- |
| No participants with serious adverse events (SAE) considered at least possibly related to administration of investigational medicinal product (IMP) i.e. a serious adverse reaction (SAR) | Escalate to the next higher dose level |
| If at least 1 on active treatment has an SAE assessed as at least possibly related to administration of IMP | 1. Stop dosing of participant with potential SAR, withdrawal of participant. 2. Unblinding participant with potential SAR, only voting members of iSRC unblinded. 3. Evaluation by iSRC and recommendation to sponsor. 4. Sponsor decision on how to proceed:  - Participant meets stop criterion, stop further dosing at this dose level for all participants - Sponsor decides if the study will be terminated or if dosing will commence at a lower dose level or at an intermediate dose level |
| **Final dosing stop and termination of study** | |
| If 2 participants on active treatment in different cohorts has SAEs assessed as at least possibly related to administration of IMP | 1. Stop dosing of participant with potential SAR, withdrawal of participant. 2. Unblinding participant with potential SAR, only voting members of iSRC unblinded. 3. Evaluation by iSRC and recommendation to sponsor. 4. Participants meet stop criterion: Termination of study. |

# Local tolerability

The incidence of local reactions, compared with baseline, was assessed by the investigator on Days 2, 3, 5, 8, 11, and 15. The investigator evaluated the incidence of the following abnormal reactions of the wound after removal of the dressing by direct inspection, prior to application of gel on treatment application days: skin and wound erythema (abnormal reaction noted); skin and wound edema and swelling (abnormal reaction noted); wound necrosis, crusting, and hemorrhage (abnormal reactions noted); and wound purulent discharge, as a sign of excessive bacterial colonization and/or infection. For each parameter, the investigator determined whether the reaction was as expected (yes/no) at each time point. Any abnormal local reactions that were reported here were also recorded as an AE, as described above.

# ECG, vital signs, and safety laboratory parameters

Clinically significant changes from baseline in ECGs, vital signs, safety laboratory parameters, and physical examinations were analyzed. Single 12-lead ECGs were recorded in supine position on an ECG machine after 10 minutes of rest. Heart rate and PR, QRS, QT, and QTcF intervals were recorded at the screening and on Day 11. ECGs were reviewed and interpreted on site by the investigator, and abnormal postdose findings that were determined by the investigator as being clinically significant were reported as AEs.

Vital signs (systolic and diastolic blood pressure and pulse) were measured using standard clinical methods while the participant was in the sitting position after 10 minutes of rest at the screening and on Day 11. Post-treatment vital signs that were judged to be “abnormal, clinically significant” by the investigator were reported as AEs.

A physical examination, consisting of assessments of general condition and the lymph nodes, throat, heart, lungs, and abdomen, was carried out at the screening and on Day 11. Abnormal postdose findings that were judged by the investigator to be clinically significant were reported as AEs.

Blood samples for analysis of clinical chemistry and hematology were collected through venepuncture or an indwelling venous catheter at the screening and on Days 2, 3, 5, and 11; sent to local hospital laboratories; and analyzed per routine analytical methods. With regard to clinical chemistry, alanine aminotransferase (ALT), aspartate aminotransferase (AST), creatinine, C-reactive protein (CRP), glucose, and hemoglobin A1c (HbA1c) were measured. Hematological parameters included hematocrite, hemoglobin (Hb), total leukocytes and differentials, thrombocytes, mean corpuscular volume (MCV), and mean corpuscular hemoglobin (MCH). Activated partial thromboplasmin time (APTT) and prothrombin complex/international normalized ratio (PK/INR) were recorded as coagulation markers. Abnormal values that were assessed by the investigator as being clinically significant were reported as AEs. If an abnormal value was associated with corresponding clinical signs or symptoms, the sign or symptom was reported as the AE.

# Definition of causality of adverse events

The causal relationship with the treatment was defined as follows:

| **Probable** | The event has a strong temporal relationship to the TCP-25 gel/procedure or recurs on re-challenge, and another etiology is unlikely or significantly less likely. |
| --- | --- |
| **Possible** | The event has a suggestive temporal relationship to the TCP-25 gel/procedure, and an alternative etiology is equally or less likely. |
| **Unlikely** | The event has no temporal relationship to the TCP-25 gel/procedure or is due to underlying/concurrent illness or effect of another drug (that is, there is no causal relationship between the TCP-25 gel/procedure and the event). |

# Adverse events

Table S2***.*** Overview of adverse events (AEs)

|  | TCP-25 0.86 mg/mL / Placebo (N=8) | | TCP-25 2.9 mg/mL / Placebo (N=8) | | TCP-25 8.6 mg/mL / Placebo (N=8) | | Total (N=24) | |
| --- | --- | --- | --- | --- | --- | --- | --- | --- |
|  | n (%) | m | n (%) | m | n (%) | m | n (%) | m |
| **Any AE** | 7 (88%) | 18 | 7 (88%) | 26 | 7 (88%) | 17 | 21 (88%) | 61 |
| **Any severe AE** | 0 | 0 | 0 | 0 | 0 | 0 | 0 | 0 |
| **Any AE leading to withdrawal from study** | 0 | 0 | 0 | 0 | 0 | 0 | 0 | 0 |
| **Any AE leading to death** | 0 | 0 | 0 | 0 | 0 | 0 | 0 | 0 |
| **Causality** |  |  |  |  |  |  |  |  |
| Unlikely Related | 7 (88%) | 18 | 7 (88%) | 26 | 7 (88%) | 17 | 21 (88%) | 61 |
| Possibly Related | 0 | 0 | 0 | 0 | 0 | 0 | 0 | 0 |
| Probably Related | 0 | 0 | 0 | 0 | 0 | 0 | 0 | 0 |
| **Severity** |  |  |  |  |  |  |  |  |
| Mild | 7 (88%) | 16 | 6 (75%) | 18 | 5 (63%) | 12 | 18 (75%) | 46 |
| Moderate | 2 (25%) | 2 | 6 (75%) | 8 | 4 (50%) | 5 | 12 (50%) | 15 |
| Severe | 0 | 0 | 0 | 0 | 0 | 0 | 0 | 0 |
| Life-Threatening | 0 | 0 | 0 | 0 | 0 | 0 | 0 | 0 |
| Death | 0 | 0 | 0 | 0 | 0 | 0 | 0 | 0 |
|  | | | | | | | | |

N: number of participants in treatment group. Percentages are based on N. n: number of participants. m: number of events.

Table S3. Adverse events by system organ class and preferred term

|  | TCP-25 0.86 mg/mL / Placebo (N=8) | | TCP-25 2.9 mg/mL / Placebo (N=8) | | TCP-25 8.6 mg/mL / Placebo (N=8) | | Total (N=24) | |
| --- | --- | --- | --- | --- | --- | --- | --- | --- |
| System organ class  Preferred term | n (%) | m | n (%) | m | n (%) | m | n (%) | m |
| **Total** | **7 (88%)** | **18** | **7 (88%)** | **26** | **7 (88%)** | **17** | **21 (88%)** | **61** |
| **Skin and subcutaneous tissue disorders** | **6 (75%)** | **8** | **5 (63%)** | **9** | **3 (38%)** | **7** | **14 (58%)** | **24** |
| Erythema | 0 | 0 | 0 | 0 | 2 (25%) | 3 | 2 (8.3%) | 3 |
| Skin irritation | 6 (75%) | 8 | 5 (63%) | 9 | 3 (38%) | 4 | 14 (58%) | 21 |
| **Infections and infestations** | **0** | **0** | **3 (38%)** | **4** | **3 (38%)** | **5** | **6 (25%)** | **9** |
| Folliculitis | 0 | 0 | 2 (25%) | 2 | 3 (38%) | 4 | 5 (21%) | 6 |
| Nasopharyngitis | 0 | 0 | 2 (25%) | 2 | 1 (13%) | 1 | 3 (13%) | 3 |
| **Nervous system disorders** | **1 (13%)** | **1** | **4 (50%)** | **5** | **1 (13%)** | **1** | **6 (25%)** | **7** |
| Dizziness | 0 | 0 | 1 (13%) | 1 | 0 | 0 | 1 (4.2%) | 1 |
| Headache | 1 (13%) | 1 | 3 (38%) | 3 | 1 (13%) | 1 | 5 (21%) | 5 |
| Paraesthesia | 0 | 0 | 1 (13%) | 1 | 0 | 0 | 1 (4.2%) | 1 |
| **Gastrointestinal disorders** | **2 (25%)** | **2** | **2 (25%)** | **2** | **1 (13%)** | **1** | **5 (21%)** | **5** |
| Abdominal pain | 1 (13%) | 1 | 1 (13%) | 1 | 0 | 0 | 2 (8.3%) | 2 |
| Abdominal pain upper | 0 | 0 | 0 | 0 | 1 (13%) | 1 | 1 (4.2%) | 1 |
| Nausea | 1 (13%) | 1 | 1 (13%) | 1 | 0 | 0 | 2 (8.3%) | 2 |
| **Respiratory, thoracic, and mediastinal disorders** | **2 (25%)** | **2** | **3 (38%)** | **4** | **0** | **0** | **5 (21%)** | **6** |
| Dysphonia | 1 (13%) | 1 | 2 (25%) | 2 | 0 | 0 | 3 (13%) | 3 |
| Nasal congestion | 1 (13%) | 1 | 0 | 0 | 0 | 0 | 1 (4.2%) | 1 |
| Oropharyngeal pain | 0 | 0 | 2 (25%) | 2 | 0 | 0 | 2 (8.3%) | 2 |
| **Musculoskeletal and connective tissue disorders** | **2 (25%)** | **3** | **1 (13%)** | **1** | **0** | **0** | **3 (13%)** | **4** |
| Arthralgia | 1 (13%) | 1 | 0 | 0 | 0 | 0 | 1 (4.2%) | 1 |
| Limb discomfort | 0 | 0 | 1 (13%) | 1 | 0 | 0 | 1 (4.2%) | 1 |
| Myalgia | 1 (13%) | 1 | 0 | 0 | 0 | 0 | 1 (4.2%) | 1 |
| Pain in extremity | 1 (13%) | 1 | 0 | 0 | 0 | 0 | 1 (4.2%) | 1 |
| **Blood and lymphatic system disorders** | **1 (13%)** | **1** | **0** | **0** | **1 (13%)** | **1** | **2 (8.3%)** | **2** |
| Anaemia | 1 (13%) | 1 | 0 | 0 | 0 | 0 | 1 (4.2%) | 1 |
| Thrombocytopenia | 0 | 0 | 0 | 0 | 1 (13%) | 1 | 1 (4.2%) | 1 |
| **Reproductive system and breast disorders** | **1 (13%)** | **1** | **1 (13%)** | **1** | **0** | **0** | **2 (8.3%)** | **2** |
| Dysmenorrhoea | 1 (13%) | 1 | 1 (13%) | 1 | 0 | 0 | 2 (8.3%) | 2 |
| **Eye disorders** | **0** | **0** | **0** | **0** | **1 (13%)** | **1** | **1 (4.2%)** | **1** |
| Eye irritation | 0 | 0 | 0 | 0 | 1 (13%) | 1 | 1 (4.2%) | 1 |
| **Vascular disorders** | **0** | **0** | **0** | **0** | **1 (13%)** | **1** | **1 (4.2%)** | **1** |
| Hemorrhage | 0 | 0 | 0 | 0 | 1 (13%) | 1 | 1 (4.2%) | 1 |
|  | | | | | | | | |

N: number of participants in treatment group. Percentages are based on N. n: number of participants. m: number of events.

Table S4. Wound-associated adverse events (AEs)

| **Dose group** | **Participant ID** | **Wound** | **Treatment** | **AE(s), verbatim text (not associated with individual wounds)** | **AE(s) PT** |
| --- | --- | --- | --- | --- | --- |
| TCP-25 0.86  mg/mL | 1101 | L1 | TCP-25 | Skin irritation bilateral thigh due to outer Tegaderm | Skin irritation |
|  |  | L2 | Placebo |  |  |
|  |  | R1 | Placebo |  |  |
|  |  | R2 | TCP-25 |  |  |
|  | 1102 | L1 | Placebo | Under outer Tegaderm skin irritation bilateral thigh | Skin irritation |
|  |  | L2 | TCP-25 |  |  |
|  |  | R1 | TCP-25 |  |  |
|  |  | R2 | Placebo |  |  |
|  | 1103 | L1 | TCP-25 | No wound-associated AE | |
|  |  | L2 | Placebo |  |  |
|  |  | R1 | Placebo |  |  |
|  |  | R2 | TCP-25 |  |  |
|  | 1104 | L1 | Placebo | Skin irritation bilateral thighs under outer Tegaderm  Skin irritation right thigh outside outer Tegaderm | Skin irritation  (2 events) |
|  |  | L2 | TCP-25 |  |  |
|  |  | R1 | TCP-25 |  |  |
|  |  | R2 | Placebo |  |  |
|  | 1105 | L1 | Placebo | Skin irritation due to Mepilex border, 2 events | Skin irritation  (2 events) |
|  |  | L2 | TCP-25 |  |  |
|  |  | R1 | TCP-25 |  |  |
|  |  | R2 | Placebo |  |  |
|  | 1106 | L1 | TCP-25 | No AE | |
|  |  | L2 | Placebo |  |  |
|  |  | R1 | Placebo |  |  |
|  |  | R2 | TCP-25 |  |  |
|  | 1107 | L1 | TCP-25 | Skin irritation bilateral thighs due to outer Tegaderm | Skin irritation |
|  |  | L2 | Placebo |  |  |
|  |  | R1 | Placebo |  |  |
|  |  | R2 | TCP-25 |  |  |
|  | 1108 | L1 | Placebo | Skin irritation right thigh under outer Tegaderm | Skin irritation |
|  |  | L2 | TCP-25 |  |  |
|  |  | R1 | TCP-25 |  |  |
|  |  | R2 | Placebo |  |  |
| TCP-25 2.9 mg/mL | 1201 | L1 | TCP-25 | Bilateral lateral skin irritation under outer Tegaderm  Bilateral skin irritation under all inner Tegaderm | Skin irritation (2 events) |
|  |  | L2 | Placebo |  |  |
|  |  | R1 | Placebo |  |  |
|  |  | R2 | TCP-25 |  |  |
|  | 1202 | L1 | TCP-25 | Folliculitis under gauze border L1  Skin irritation under Tegaderm (outer) right thigh medial  Skin irritation under outer Tegaderm-border craniomedial bilateral thigh | Folliculitis Skin irritation (2 events) |
|  |  | L2 | Placebo |  |  |
|  |  | R1 | Placebo |  |  |
|  |  | R2 | TCP-25 |  |  |
|  | 1203 | L1 | Placebo | No AE | |
|  |  | L2 | TCP-25 |  |  |
|  |  | R1 | TCP-25 |  |  |
|  |  | R2 | Placebo |  |  |
|  | 1204 | L1 | Placebo | No wound-associated AE | |
|  |  | L2 | TCP-25 |  |  |
|  |  | R1 | TCP-25 |  |  |
|  |  | R2 | Placebo |  |  |
|  | 1205 | L1 | Placebo | No wound-associated AE | |
|  |  | L2 | TCP-25 |  |  |
|  |  | R1 | TCP-25 |  |  |
|  |  | R2 | Placebo |  |  |
|  | 1206 | L1 | TCP-25 | Folliculitis along border of Mepilex L1 and L2  Skin irritation along position of outer Tegaderm border medial right thigh  Skin irritation along Mepilex border at R2 and L2 | Folliculitis Skin irritation (2 events) |
|  |  | L2 | Placebo |  |  |
|  |  | R1 | Placebo |  |  |
|  |  | R2 | TCP-25 |  |  |
|  | 1207 | L1 | TCP-25 | Skin irritation under all inner Tegaderm  Skin irritation under outer Tegaderm bilateral thigh | Skin irritation (2 events) |
|  |  | L2 | Placebo |  |  |
|  |  | R1 | Placebo |  |  |
|  |  | R2 | TCP-25 |  |  |
|  | 1208 | L1 | Placebo | Skin irritation along outer border of outer Tegaderm craniolateral bilateral | Skin irritation |
|  |  | L2 | TCP-25 |  |  |
|  |  | R1 | TCP-25 |  |  |
|  |  | R2 | Placebo |  |  |
| TCP-25 8.6 mg/mL | 1301 | L1 | TCP-25 | General redness under borders of outer Tegaderm bilateral  General redness under borders of inner Tegaderm bilateral  Skin irritation around all Mepilex borders | Erythema (2 events)  Skin irritation |
|  |  | L2 | Placebo |  |  |
|  |  | R1 | Placebo |  |  |
|  |  | R2 | TCP-25 |  |  |
|  | 1302 | L1 | Placebo | No AEs | |
|  |  | L2 | TCP-25 |  |  |
|  |  | R1 | TCP-25 |  |  |
|  |  | R2 | Placebo |  |  |
|  | 1303 | L1 | Placebo | Skin irritation around all borders of Mepilex | Skin irritation |
|  |  | L2 | TCP-25 |  |  |
|  |  | R1 | TCP-25 |  |  |
|  |  | R2 | Placebo |  |  |
|  | 1304 | L1 | TCP-25 | Folliculitis under all inner Tegaderm  Folliculitis under outer Tegaderm bilateral | Folliculitis (2 events) |
|  |  | L2 | Placebo |  |  |
|  |  | R1 | Placebo |  |  |
|  |  | R2 | TCP-25 |  |  |
|  | 1305 | L1 | TCP-25 | No wound-associated AE | |
|  |  | L2 | Placebo |  |  |
|  |  | R1 | Placebo |  |  |
|  |  | R2 | TCP-25 |  |  |
|  | 1306 | L1 | TCP-25 | Folliculitis under outer Tegaderm bilateral | Folliculitis |
|  |  | L2 | Placebo |  |  |
|  |  | R1 | Placebo |  |  |
|  |  | R2 | TCP-25 |  |  |
|  | 1307 | L1 | Placebo | No wound-associated AE | |
|  |  | L2 | TCP-25 |  |  |
|  |  | R1 | TCP-25 |  |  |
|  |  | R2 | Placebo |  |  |
|  | 1308 | L1 | Placebo | Skin erythema under inner Tegaderm of R1, R2 & L2  Folliculitis around Mepilex border of L2  Previous bleeding L2 ( Mepilex saturated with blood)  Skin irritation around border of Mepilex R1  Skin irritation under border of all inner Tegaderm | Erythema  Hemorrhage  Skin irritation (2 events) |
|  |  | L2 | TCP-25 |  |  |
|  |  | R1 | TCP-25 |  |  |
|  |  | R2 | Placebo |  |  |

| Table S5. Non-wound-associated adverse events (AEs) | | | | | | |
| --- | --- | --- | --- | --- | --- | --- |
| **TCP-25 Dose** | **Participant ID** | **Reported Term** | **Standard Toxicity Grade*** | **Study Day of Start of AE** | **Study Day of End of AE** | **Outcome of AE** |
| 0.86 mg/mL | 1101 | Hoarseness | MODERATE | 14 | NA | NOT RESOLVED |
|  |  | Anemia | MILD | 11 | NA | NOT RESOLVED |
|  | 1102 | Pain hand bilateral | MILD | 11 | NA | NOT RESOLVED |
|  |  | Wrist pain bilateral | MILD | 11 | NA | NOT RESOLVED |
|  | 1103 | Headache | MILD | 2 | 2 | RESOLVED |
|  | 1105 | Nausea | MILD | 2 | 2 | RESOLVED |
|  |  | Nasal congestion | MILD | 7 | 7 | RESOLVED |
|  | 1108 | Muscle ache | MILD | 8 | NA | NOT RESOLVED |
|  |  | Dysmenorrhea | MODERATE | 9 | 13 | RESOLVED |
|  |  | Abdominal cramp | MILD | 9 | NA | NOT RESOLVED |
| 2.9 mg/mL | 1201 | Dizziness | MILD | 1 | 1 | RESOLVED |
|  |  | Nausea | MODERATE | 1 | 1 | RESOLVED |
|  |  | Hoarseness | MILD | 5 | 11 | RESOLVED |
|  |  | Common cold | MILD | 8 | NA | NOT RESOLVED |
|  | 1202 | Paresthesia left arm | MODERATE | 4 | 5 | RESOLVED |
|  |  | Headache | MODERATE | 6 | 6 | RESOLVED |
|  |  | Common cold | MILD | 12 | NA | NOT RESOLVED |
|  | 1204 | Discomfort left leg | MODERATE | 14 | NA | NOT RESOLVED |
|  |  | Sore throat | MODERATE | 9 | 10 | RESOLVED |
|  | 1205 | Headache | MODERATE | 9 | 10 | RESOLVED |
|  | 1206 | Abdominal pain | MILD | 6 | NA | RESOLVING |
|  |  | Headache | MODERATE | 12 | 12 | RESOLVED |
|  | 1207 | Period pain | MODERATE | 12 | 13 | RESOLVED |
|  | 1208 | Sore throat | MILD | 3 | 4 | RESOLVED |
|  |  | Hoarseness | MILD | 6 | NA | RESOLVING |
| 8.6 mg/mL | 1304 | Stomachache | MODERATE | 4 | 4 | RESOLVED |
|  | 1305 | Headache | MODERATE | 7 | 7 | RESOLVED |
|  | 1307 | Thrombocytopenia | MODERATE | 11 | NA | NOT RESOLVED |
|  | 1308 | Common cold | MILD | 7 | NA | RESOLVING |
|  |  | Irritation of left eye | MILD | 11 | 15 | RESOLVED |

NA: Not applicable.

*****: All AEs were judged by the investigator to be unlikely related to the study drug.

#
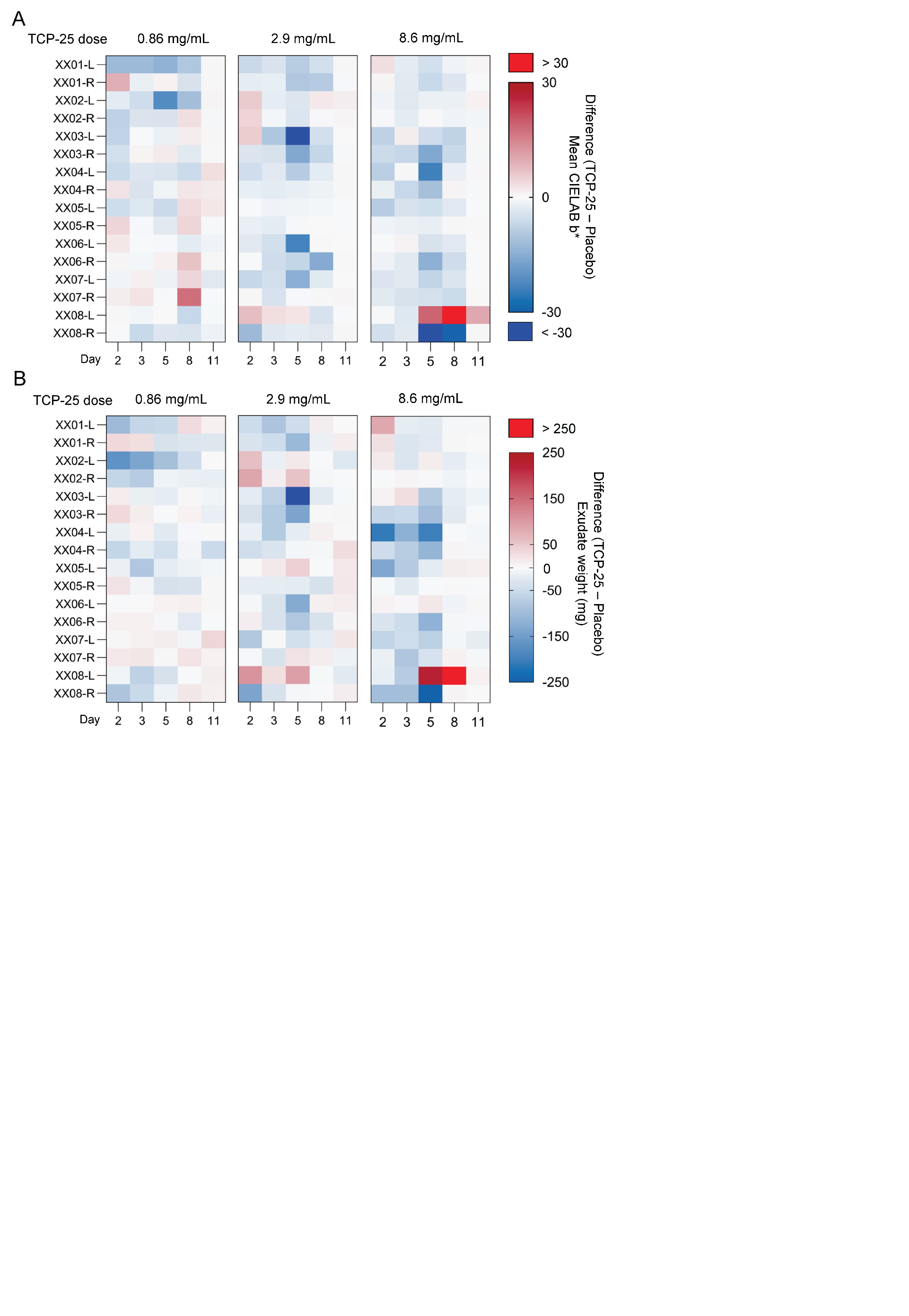
Exploratory analysis

Figure S1**.** Heat map analysis of individual wound pair differences in exudation measures.
(A-B) Heat maps display within-participant differences in dressing yellowness (CIELAB b*) and exudation weight between TCP-25-treated and placebo-treated wounds across all timepoints (days 2, 5, 8, and 11). Each row represents one wound pair (left and right leg wounds) from a single participant (n=16 pairs from 8 participants per dose group). Blue coloring indicates lower values in TCP-25-treated wounds compared to placebo; red indicates higher values. TCP-25-treated wounds typically showed reduced dressing yellowness (A) and exudation weight (B) compared to placebo in wound pairs, with more pronounced reductions in the 2.9 and 8.6 mg/mL TCP-25 dose groups. Minimal differences were observed on day 11 across all dose groups.


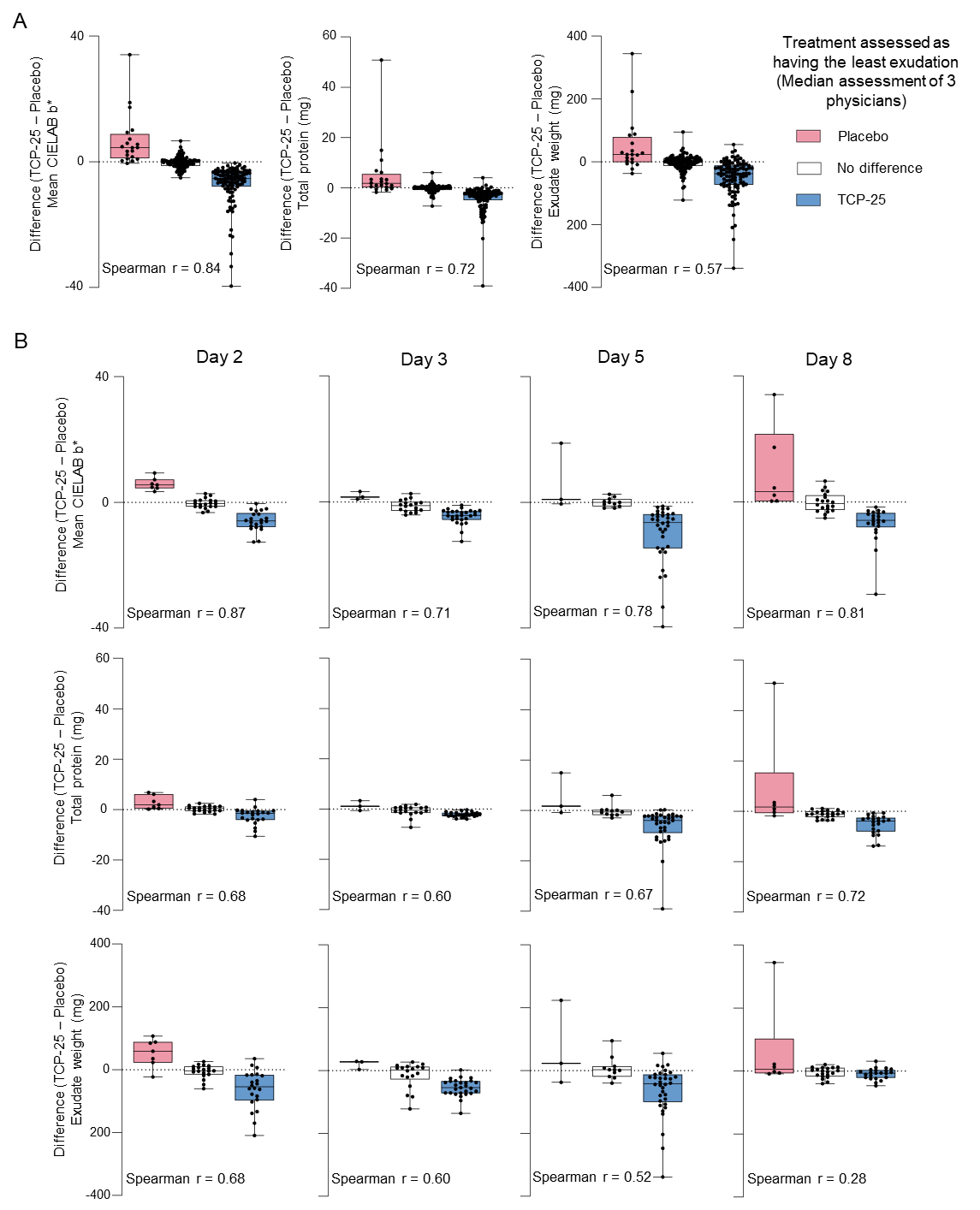


Figure S2**.** Correlation between manual visual assessment and difference in quantitative exudation measures. (A-B) Each point represents one wound pair. Three blinded physicians independently determined, for each wound pair, whether the TCP-25 treated, the placebo treated, or neither had the least visible signs of exudation in dressing images. The color denotes the median result of the three assessors. The y-axis shows the corresponding difference (TCP-25 minus placebo) for each quantitative measure. (A) Pooled analysis across all dose groups and timepoints (n=240 wound-pair measurements; 48 pairs from 24 participants at 5 timepoints). (B) Time-specific analysis for days 2, 3, 5, and 8 (day 11 excluded due to all pairs but one being scored as equal). Correlation patterns were generally consistent with pooled results, with the strongest correlations observed for dressing yellowness (r = 0.71 – 0.87) and total protein content (r = 0.60 – 0.72). Exudation weight correlations were more variable across timepoints (r = 0.28 – 0.68), with notably weaker correlation on day 8. n = 48 wound pairs from 24 participants at the indicated time points.


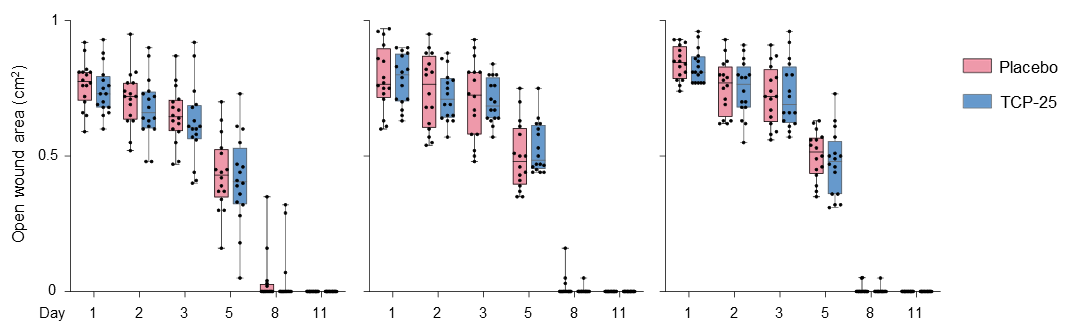


Figure S3**.** Planimetric analysis of open wound area over time from digital wound images. Box plots show median (center line), interquartile range (box), and minimum-maximum values (whiskers); individual data points represent single wound (n=32 wounds from 8 participants per dose group and timepoint). The color denotes the treatment allocation. The results suggest comparable wound size at Day 1 prior to treatment. Open wound area decreased over time in both groups, with the majority of wounds closed by Day 8 and all closed by Day 11.

# References

1. Papareddy P, Rydengård V, Pasupuleti M, Walse B, Mörgelin M, Chalupka A, et al. Proteolysis of human thrombin generates novel host defense peptides. PLoS Pathog. 2010;6(4):e1000857.

2. Puthia M, Butrym M, Petrlova J, Stromdahl AC, Andersson MA, Kjellstrom S, et al. A dual-action peptide-containing hydrogel targets wound infection and inflammation. Sci Transl Med. 2020;12(524):eaax6601.

3. Saravanan R, Holdbrook DA, Petrlova J, Singh S, Berglund NA, Choong YK, et al. Structural basis for endotoxin neutralisation and anti-inflammatory activity of thrombin-derived C-terminal peptides. Nat Commun. 2018;9(1):2762.

4. Kalle M, Papareddy P, Kasetty G, Mörgelin M, van der Plas MJ, Rydengård V, et al. Host defense peptides of thrombin modulate inflammation and coagulation in endotoxin-mediated shock and Pseudomonas aeruginosa sepsis. PLoS One. 2012;7(12):e51313.

5. Xinnate AB. TCP-25 Toxicology Studies (unpublished data on file). Lund, Sweden.
