# Supplementary material for: A First‐In‐Human Randomized Controlled Phase 1 Study Assessing the Safety and Tolerability of Topical TCP‐25 Gel in Epidermal Suction Blister Wounds
Source: Clin Transl Sci. 2026 Feb 5;19(2):e70497. doi: 10.1111/cts.70497 (PMC12877310; doi:10.1111/cts.70497)
Supplement: Supplementary file 3 — Data S3: cts70497‐sup‐0003‐supinfo.pdf. [file CTS-19-e70497-s001.pdf]

1 STATISTICAL METHODS AND DETERMINATION OF SAMPLE SIZE

The principal features of the statistical analysis to be performed are described in this section.

Analyses of the primary and secondary endpoints will be performed by CTC.

1.1 General

Continuous data will be presented in terms of evaluable and missing observations, arithmetic mean, standard deviation (SD), median, minimum and maximum value.

Categorical data will be presented as counts and percentages. When applicable, summary data will be presented by treatment, and by assessment time. Individual subject data will be listed by subject number, treatment, and, where applicable, by assessment time.

All descriptive summaries and statistical analyses will be performed using SAS Version 9.4 or later (SAS Institute, Inc., Cary, NC).

Baseline will be defined as the last non-missing observation prior to the first administration of IMP.

Handling of dropouts, missing data and outliers

Outliers will be included in summary tables and listings and will not be handled separately in any analyses. Generally, no imputation of data will be performed. However, clinical safety laboratory parameters that are outside the detection limit, will be replaced with the detection limit when calculating statistics. Also, when calculating statistics for PK plasma concentrations, concentrations under LLOQ will be replaced with LLOQ if more than 50% of the values for a given time point is above LLOQ. Otherwise, no statistics will be calculated for that time point.

Output rounding principles

Generally, no rounding of data will be done prior to calculating statistics. However, if reported data contains more than 8 significant digits it will be rounded to 8 significant digits in the database.

In statistical output and descriptive summaries, the following principles will be followed:

- 3 significant digits will be used for PK and similar parameters.
- 2 significant digits will be used for percentages (for example relative change from baseline).
- p-values and similar statistical output will be presented using 4 decimal points.

All descriptive summaries of numerical data (e.g., mean, SD etc.) will be presented with one extra decimal compared to reported input data.

1.2 Determination of sample size

No formal sample size calculation has been performed for this study. The proposed sample size is considered sufficient to provide adequate information for the study objectives.

Approximately 50 subjects will be screened to achieve 24 randomized and evaluable subjects.

1.3 Analysis data sets

17.3.1 *Full analysis set*

The Full Analysis Set (FAS) will consist of all subjects who have been randomized and received at least one dose of IMP and who has at least one post-baseline assessment of data.

1.4 Description of study population

17.4.1 *Demographics and baseline characteristics*

Descriptive statistics for demographics, weight and height will be presented by treatment and dose group.

17.4.2 *Medical/surgical history and prior/concomitant medication*

Medical/surgical history will be presented by system-organ-class (SOC) and preferred term (PT). Prior/concomitant medications will be presented by ATC level 1, 3 and 5.

All data will be listed by subject

17.4.3 *Treatment compliance*

The number of subjects treated in each dose group and their individual dose will be listed.

The compliance in will be presented using summary statistics per dose group.

1.5 Analysis of primary endpoints

17.5.1 *Adverse events*

An overview of all AEs, including SAEs, intensity, relationship to IMP, and deaths will be presented by SOC and PT.

Incidence of AEs and SAEs will be summarised by SOC and PT by treatment, dose group and overall.

All AE data will be listed subject and include the verbatim term entered by the Investigator.

17.5.2 *Local tolerability*

The following parameters for incidence of abnormal local reactions as compared to wound healing will be directly assessed by the Investigator:

- Skin and wound erythema (abnormal reaction noted)
- Skin and wound oedema and swelling (abnormal reaction noted)
- Wound necrosis, crusting, and hemorrhage (abnormal reactions noted)
- Wound purulent discharge as sign of excessive bacterial colonization and/or infection (abnormal reactions noted)

Each variable will be summarised using descriptive statistics by dose group, treatment (active/placebo) and visit, as applicable.

All data will be listed by dose group, treatment, wound location, visit and subject.

17.5.3 *12-lead ECG*

All ECGs will be categorised as ”normal”, ”abnormal, not clinically significant”, or ”abnormal, clinically significant” (as judged by the Investigator) and summarised by treatment and dose group using frequency tables.

Changes over time will be presented using shift tables, if considered appropriate.

All data will be listed by subject.

17.5.4 *Vital signs*

Vital signs (systolic/diastolic blood pressure and pulse) will be summarised by treatment and dose group. Data will be presented with absolute and percent change from baseline.

All data will be listed by subject.

17.5.5 *Safety laboratory analyses*

Safety laboratory data will be summarised by treatment and dose group with absolute and percent change from baseline at each visit.

Abnormal, clinically significant values will be summarised separately, if considered appropriate.

All data will be listed by subject.

17.5.6 *Physical examination*

Clinically significant and non-clinically significant abnormal findings will be specified and presented by subject and summarised by treatment and dose group.

Changes over time will be presented using shift tables, if considered appropriate.

All data will be listed by subject.

1.6 Analysis of secondary endpoints

17.6.1 *Analysis of plasma concentrations of TCP-25*

Summary statistics for the plasma concentrations of TCP-25 will be presented by treatment and dose group with number of measurements, arithmetic mean, SD, coefficient of variation (CV), median, minimum, maximum. All plasma concentration data will be listed by subject.

In case of a measurable systemic exposure to TCP-25, a PK analysis will be performed, and relevant PK parameters may be calculated, if possible. The PK parameters will in such case be calculated by non-compartmental analysis (NCA) using the software Phoenix WinNonlin® version 8.1 or later (Certara, U.S.A.).

1.7 Analysis of exploratory endpoints

Details of the analysis will be given elsewhere, and the data will not form a part of the CSR.
